# High-performance Supercapacitors Based on Electrochemical-induced Vertical-aligned Carbon Nanotubes and Polyaniline Nanocomposite Electrodes

**DOI:** 10.1038/srep43676

**Published:** 2017-03-08

**Authors:** Guan Wu, Pengfeng Tan, Dongxing Wang, Zhe Li, Lu Peng, Ying Hu, Caifeng Wang, Wei Zhu, Su Chen, Wei Chen

**Affiliations:** 1 The State Key Laboratory of Materials-Oriented Chemical Engineering and College of Chemistry and Chemical Engineering, Nanjing Tech University, Nanjing 210009, P. R. China; 2 i-Lab, Suzhou Institute of Nano-tech and Nano-bionics, Chinese Academy of Sciences, Suzhou 215123, P. R. China; 3 Institute of Industry and Equipment Technology, Hefei University of Technology, Hefei, Anhui 230009, P. R. China

## Abstract

Supercapacitors, which store electrical energy through reversible ion on the surface of conductive electrodes have gained enormous attention for variously portable energy storage devices. Since the capacitive performance is mainly determined by the structural and electrochemical properties of electrodes, the electrodes become more crucial to higher performance. However, due to the disordered microstructure and low electrochemical activity of electrode for ion tortuous migration and accumulation, the supercapacitors present relatively low capacitance and energy density. Here we report a high-performance supercapacitor based on polyaniline/vertical-aligned carbon nanotubes (PANI/VA-CNTs) nanocomposite electrodes where the vertical-aligned-structure is formed by the electrochemical-induction (0.75 V). The supercapacitor displays large specific capacitance of 403.3 F g^−1^, which is 6 times higher than disordered CNTs in HClO_4_ electrolyte. Additionally, the supercapacitor can also present high specific capacitance (314.6 F g^−1^), excellent cycling stability (90.2% retention after 3000 cycles at 4 A g^−1^) and high energy density (98.1 Wh kg^−1^) in EMIBF_4_ organic electrolyte. The key to high-performance lies in the vertical-aligned-structure providing direct path channel for ion faster diffusion and high electrochemical capacitance of polyaniline for ion more accommodation.

Supercapacitors, which are the branch of energy storage devices have attracted significant interests in a variety of applications, including portable electronics, power supply devices and electric vehicles^1^,^2^,^3^,^4^,^5^,^6^. They stored and released electrical energy are due to the formation of electrical double layers (the reversible ion absorption at the electrode-electrolyte interface)^7^, which endow the supercapacitors excellent electrochemical performance such as high power density, long cycle lifetimes and low cost^8^,^9^,^10^,^11^,^12^,^13^. The electrochemical performances of supercapacitors are mainly controlled by the structural and electrochemical properties of electrodes materials. Nanocarbon materials such as CNTs have shown anisotropic microstructure, porous networks, high electric conductivity and large mechanical strength, which make them prime candidates as supercapacitor electrodes^5^,^14^,^15^,^16^,^17^,^18^,^19^. For instance, large-scale free-standing single-walled carbon nanotubes (SWCNTs) film with high electric conductivity were utilized as supercapacitor electrodes, and the supercapacitors performed good electrochemical performance (specific capacitance was 35 F g^−1^, energy density was 43.7 Wh kg^−1^)^20^. To improve the electrochemical performance, three-dimensional CNTs bridged graphene electrode with stable porous networks presented a high energy density of 110.6 Wh kg^−1^ 21. Furthermore, anisotropic arranged CNTs electrodes via chemical vapor deposition method enhanced the capacitance (106.2 F g^−1^) by providing smooth path channel for ion fast migration in electrodes^22^.

To further improve the capacitive performance, conducting polymers, which were served as electrochemically active materials for pseudo capacitance were introduced in CNTs^23^,^24^,^25^,^26^. Among various conducting polymer, PANI was considered to be one of the most promising active materials because of its relatively large specific pseudocapacitance, high conductivity and low cost of aniline monomers. Meanwhile, after adding PANI in CNTs, the electrochemical performance of supercapacitors would be greatly enhanced^27^,^28^. Lin *et al*. reported PANI incorporated with aligned CNTs electrodes based supercapacitors. A large specific capacitance (233 F g^−1^) was achieved owing to PANI generating large redox capacitance^29^. Xie’s group reported a “skeleton/kin” strategy to fabricate SWCNTs/PANI with controlled morphology and microstructure based supercapacitors where the supercapacitors exhibited high energy density (131 Wh kg^−1^)^30^. Ge *et al*. reported a novel supercapacitor based on SWCNTs and PANI nanoribbons interpenetrated within cellulose fiber network, which brought a fast electron transport in SWCNTs and charge transfer of PANI nanoribbons^31^. Obviously, the electrochemical performance of supercapacitors was largely enhanced by the useful process of structural and electrochemical design of electrode materials. However, almost no effective and facile approach to make the combination between the vertical-aligned-structure and electrochemical activity so that to substantially promote the electrochemical performance of supercapacitor remains a challenge.

In this work, motivated by the combination between the vertical-aligned-structure and electrochemical activity design, we develop a supercapacitor based on PANI/VA-CNTs electrodes in which the vertical-aligned-structure is obtained by a simply electrochemical induction, and simultaneously the PANI is *in-situ* grown on the CNTs. Owing to the vertical-aligned-structure providing straight and fast ion transport pathchannel, large pseudocapacitance of polyaniline and high electric conductivity, the supercapacitor displays high electrochemical performance, including large specific capacitance, good cycling stability and high energy density. Our work clarifies an important role of structural and electrochemical activity design, which will guide the development of next-generation supercapacitors.

## Results

### Fabrication and characterization of PANI/VA-CNTs

**Figure 1:**
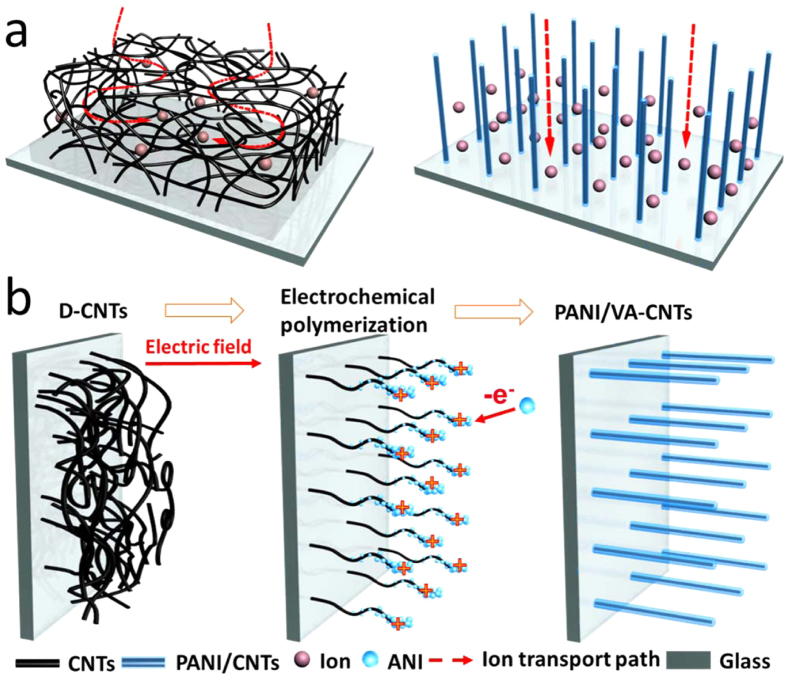
Schematic illustration of the architecture and fabrication of designed electrode. (**a**) Schematic illustration of the designed electrode structure. (**b**) Schematic illustration of preparing the PANI/VA-CNTs film.

The designed structure of supercapacitor electrode was shown in Fig. 1a. Normally, disordered CNTs (D-CNTs) were randomly aggregated and the charges (ions) had to cross a long pathway, resulting in the less charge accumulation in electrodes so that the supercapacitor performed poor capacitance. However, after the disordered structure becoming vertical alignment, the continuously conductive pathways in the VA-CNTs could allow charges straight and fast transport. Moreover, when the PANI was electropolymerized on the VA-CNTs backbone, more charges would be accommodated in the electrode through the redox reaction, which further enhanced the electrochemical performance. The synthesized process of PANI/VA-CNTs electrode was carried out via the three-electrode system of electrochemical polymerization (Figure S1). As illustrated in Fig. 1b, the system consisted of loose D-CNTs film as working electrode, Ag/AgCl reference electrode, platinum plate counter electrode and 0.1 M aniline in 1 M HClO_4_ electrolyte. On application of an electric field, the disordered CNTs was induced so that to elementary orientate along the electric field’s direction^32^,^33^. Meanwhile, the aniline monomers in electrolyte lost electron and were *in-situ* electrodeposited on the carbon nanotubes. Besides, the positive charged particle would be moved^34^,^35^ to the electric field’s direction so that CNTs was accelerate to be vertical alignment^36^, leading to the increase of CNTs film. Then, PANI would continue to grow until they filled the entire network. It is due to the synergistic effect between the electric field induction and electropolymerization of PANI that we obtained the PANI/VA-CNTs nanocomposite electrode films.

**Figure 2:**
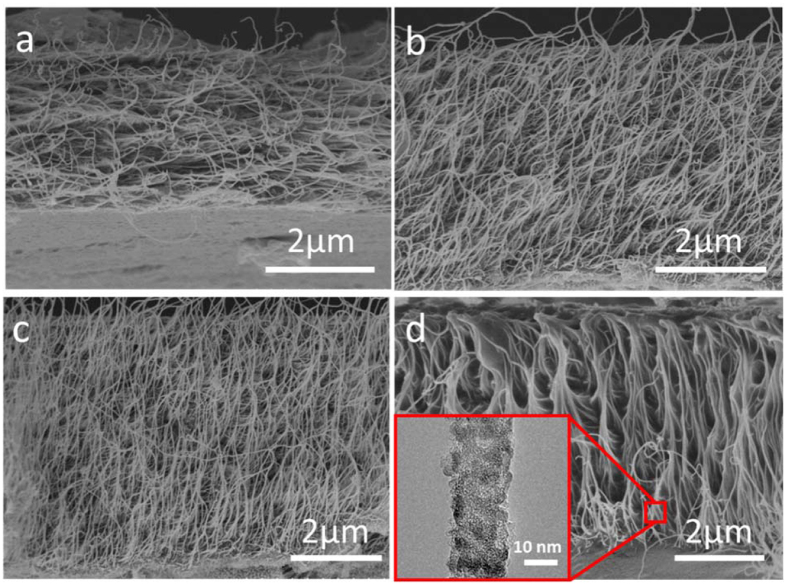
Morphology characterization of PANI/VA-CNTs nanocomposite electrode film. Cross-sectional SEM images of PANI/VA-CNTs electrodes prepared within variously electrochemical polymerization (**a**) 0 min, (**b**) 20 min, (**c**) 40 min and (**d**) 60 min, and the inset in Fig. 2d is the TEM image of PANI/CNTs.

The morphology structure of PANI/VA-CNTs was characterized by scanning electron microscopy (SEM) and transmission electron microscopy (TEM). As shown the cross-sectional image in Fig. 2a, the CNTs film presented a randomly disordered arrangement and its thickness was 2.9 μm. With the electrochemical-induced time reaching 20 min, the D-CNTs was gradually formed a sparsely vertical-aligned nanostructure and the thickness of film was increased to 4.4 μm (Fig. 2b). After the 40 min electrochemical induction, the orderly and regularly vertical alignment of PANI/CNTs was formed, which was clearly shown in Fig. 2c, and the thickness of film arrived at 5.4 μm. When the electrochemical-induced time exceeding 60 min, the PANI would be covered the whole network of carbon film and the thickness of film was maintained at 5.6 μm (Fig. 2d). The inset TEM image in Fig. 2d was further confirmed that the PANI was *in-situ* grown on carbon nanotubes, guaranteeing a good electron conduction between CNTs and PANI in the network. Raman and Fourier transform infrared (FTIR) spectra were characterized the structures of PANI/CNTs nanocomposite electrode. As shown the Raman spectrum in Figure S2, D-CNTs exhibited a strong G band peak at 1592 cm^−1^, corresponded to the in-plane stretching E_2g_ phonons^37^. After electro-polymerizing PANI on CNTs, the peaks at 1169 cm^−1^ and 1337 cm^−1^ were ascribed to C-H bending of benzene ring and C-N stretching of benzene ring. The peaks at 1491 cm^−1^, 1616 cm^−1^ were assigned to C = C stretching of benzene ring and C = C stretching of quinonoid ring^37^. Furthermore, the FTIR spectrum of PANI/CNTs in Figure S3 showed a typical group bands of C = C stretching vibration of the quinonoid ring and benzenoid ring at 1558 cm^−1^ and 1475 cm^−1^, respectively and C–N vibrations of secondary aromatic amines at of PANI 1290 cm^−1^ 11,38,39.

### Electrochemical performance of PANI/VA-CNTs in aqueous electrolyte

**Figure 3:**
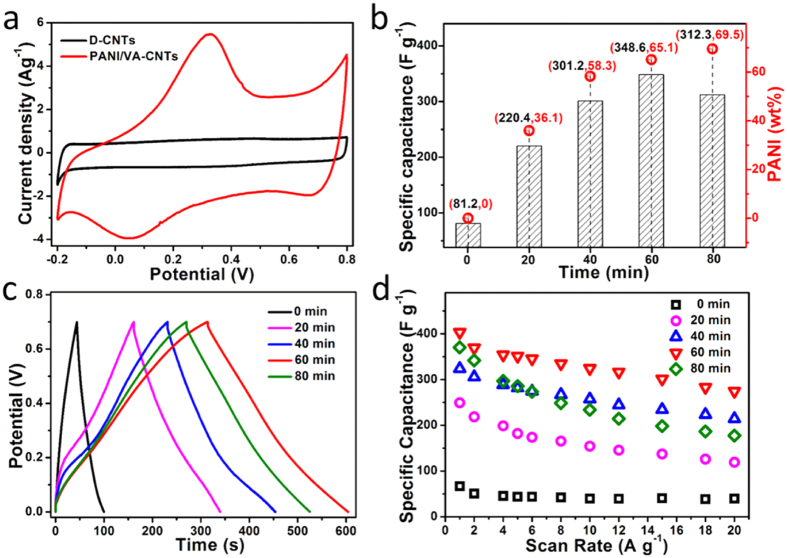
Electrochemical characterization of the PANI/VA-CNTs in 1 M HClO_4_. (**a**) CV curves of PANI/VA-CNTs and D-CNTs at a scan rate of 10 mV s^−1^. (**b**) The calculated specific capacitances and PANI weight percentages in PANI/VA-CNTs electrodes under variously electrochemical-induced time at a scan rate of 10 mV s^−1^. (**c**) Galvanostatic charge/discharge curves of PANI/VA-CNTs under variously electrochemical-induced time at a current density of 1 A g^−1^. (**d**) The calculated specific capacitances under different current densities.

The cyclic voltammetry (CV) and galvanostatic cycle measurements were characterized to demonstrate the electrochemical performance of supercapacitors. After the disordered structure becoming vertical arrangement by electrochemical induction, the PANI/VA-CNTs supercapacitor obviously presented a greatly enhanced of electrochemical performance in comparison with D-CNTs. As shown the CV curves in Fig. 3a, a better charge/discharge behavior with large area and redox peaks, which revealed the pseudocapacitance originated from leucoemeraldine/emeraldine and emeraldine/pernigraniline transitions of PANI^11^, were achieved for PANI/VA-CNTs supercapacitor, while D-CNTs only exhibited a small rectangular shape of electric double layer capacitor (EDLC) at the scan rate of 10 mV s^−1^. Through controlling the electrochemical-induced time, we obtained various loading amounts of the PANI/VA-CNTs (the PANI weight percentage in nanocomposite electrode) and the corresponding specific capacitance (Fig. 3b). It was observed that the weight percentages of PANI were as follows: 36.1 wt.%, 58.3%, 65.1% and 69.5% under 20 min, 40 min, 60 min, and 80 min electrochemical induction, respectively. By optimizing the loading amount, PANI/VA-CNTs with 60 min electrochemical induction exhibited the highest capacitance (348.6 Fg^−1^).The galvanostatic charge/discharge behaviors of variously electrochemical induction supercapacitors were shown in Fig. 3c. The basically symmetric curve between charge and discharge cycles revealed the good reversibility of supercapacitors. Additionally, as revealed from charge/discharge curves, the capacitance would be raised with the electrochemical-induced time increasing from 0 min to 60 min, which were well in accordance with the wt.% changes of PANI in Fig. 3b. However the capacitance would be decreased with electrochemical-induced time exceeding 60 min because the overgrowth PANI could not only inefficiently interact with VA-CNTs but also hinder the ion diffussion^29^,^36^. The detailed specific capacitance variation was estimated in Fig. 3d. At a current density of 1 A g^−1^, PANI/VA-CNTs prepared under 60 min electrochemical induction represented the largest specific capacitance of 403.3 F g^−1^, which was more than 6 time higher than the pure D-CNTs (66.6 F g^−1^). Furthermore, at a higher current density of 20 A g^−1^, PANI/VA-CNTs could still perform a high capacitance of 274.2 F g^−1^. Precisely, it is the synergetic effect between vertically aligned CNTs and PANI, which played the key role to improve electrochemical performance of supercapacitors.

### Electrochemical performance of PANI/VA-CNTs in organic electrolyte

**Figure 4:**
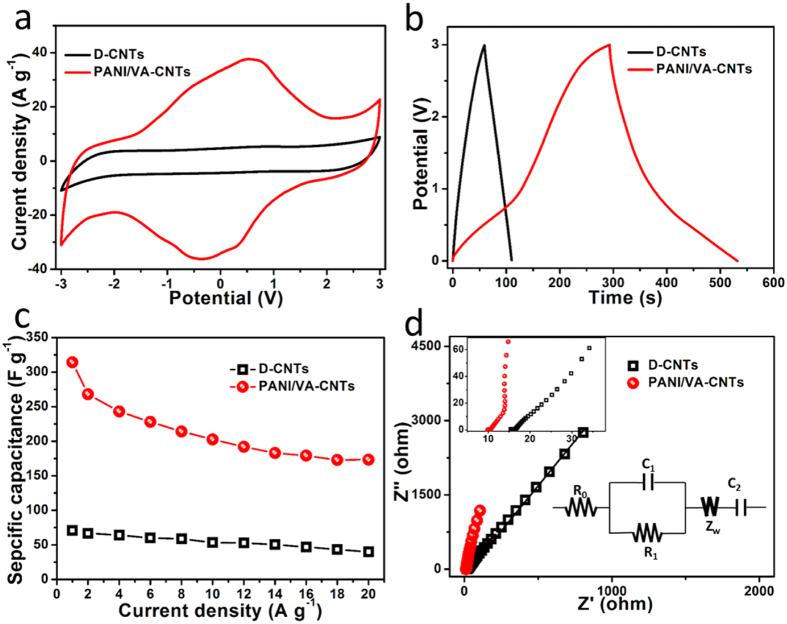
Electrochemical characterization of the PANI/VA-CNTs in 1 M EMIBF_4_/PC. (**a**) CV curves of PANI/VA-CNTs and D-CNTs at a scan rate of 100 mV s^−1^. (**b**) Galvanostatic charge/discharge curves of PANI/VA-CNTs at the current density of 1 A g^−1^. (**c**) The calculated specific capacitances under different current densities. (**d**) EIS analysis of supercapacitors. The inset figures were the depressed semicircle of Nyquist plots and the equivalent circuit model. Symbols denoted experimental data, while the continuous lines represented the fitted data.

The energy density of supercapacitor is mainly dominated by the operated potential, which is proportional to the square of voltage. To improve the stored energy, organic electrolyte was utilized because of its wider electrochemical window. The supercapacitor was constructed by two piece of as-prepared PANI/VA-CNTs nanocomposite electrodes, which were separated by a porous paper. The organic electrolyte was 1 M EMIBF_4_/PC where EMIBF_4_ was 1-eutyl-3-methylimidazolium tetrafluoroborate and PC was propylene carbonate. As shown in Fig. 4a, the PANI/VA-CNTs cell significantly showed a larger energy storage behavior under a wider potential window of −3 V to 3 V than D-CNTs at a scan rate of 100 mV s^−1^. Meanwhile, the galvanostatic charge/discharge curves of PANI/VA-CNTs cell exhibited a nearly symmetric curve, which showed small deviations from linearity owing to the pseudocapacitive feature of PANI (Fig. 4b). The specific capacitance of supercapacitors calculated from discharge curve under wide potential window was shown in Fig. 4c. It was revealed that the specific capacitance decreased with the increase of current density. At a current density of 1 A g^−1^, the specific capacitance of PANI/VA-CNTs supercapacitor was 314.6 F g^−1^, which was much higher than the D-CNTs (70.6 F g^−1^). When the current density increasing to 10 A g^−1^, only 64.4% of capacitance (202.7 F g^−1^) could be maintained for PANI/VA-CNTs and 53.3 F g^−1^ for D-CNTs (Figure S4). When the current density was increased to as high as 20 A g^−1^, the PANI/VA-CNTs could still represent a large specific capacitance of 173.3 F g^−1^, whereas the D-CNTs just preserved 40.8 F g^−1^ (Figure S5).

In order to understand electrochemical induction of PANI/VA-CNTs affecting the electrochemical behavior of supercapacitors, the electrochemical impedance spectroscopy (EIS) was carried out^23^,^36^,^40^,^41^. As shown in Fig. 4d, an equivalent circuit model was utilized to analyse the Nyquist plots. In this model, R_0_ was the internal resistance of supercapacitor, the element C_1_/R_1_ represented the contact impedance between electrode film and metal current collector, Warburg diffusion impedance Z_w_ and the elements C_2_ represented the intercalation capacitance, which was ascribed to the vertical-aligned-structure and PANI contribution. As shown the fitting parameters in Table S1, the PANI/A-CNTs exhibited a smaller R_0_ (9.6 Ω) than D-CNTs (15.3 Ω), which was resulted from the good electric conductivity of electrodes. In addition, a better EDLC and conductivity of PANI/VA-CNTs (0.94 mF/0.75 Ω) than D-CNTs (0.14 mF/2.32 Ω) when taking the C_1_/R_1_ for comparison. Furthermore, compared with the ion diffusion ability of Z_w_, a lower resistance of PANI/VA-CNTs (0.43 Ω) was obtained than D-CNTs (2.15 Ω), which was revealed that the vertical-aligned-structure provided open pathways for ion faster diffusion. When taking C_2_ into account, PANI/VA-CNTs performed larger intercalation capacitance (18.3 mF) than D-CNTs (2.74 mF). It could be found that PANI/VA-CNTs was effectively improved the electrochemical performance of supercapacitor.

**Figure 5:**
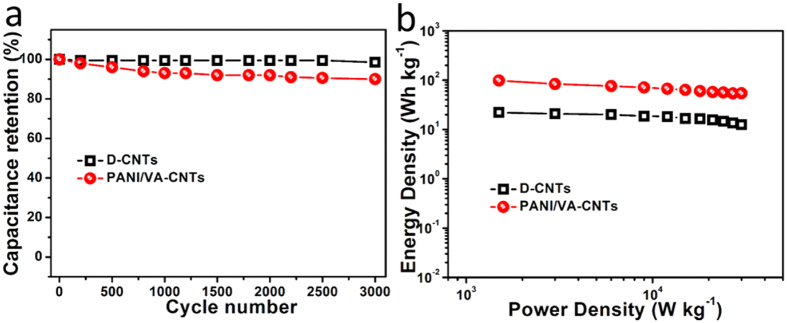
The cyclic stability and energy stored in supercapacitors. (**a**) Cycle testing of supercapacitors under a voltage of 3 V at a current density of 4 A g^−1^. (**b**) Energy density versus power density of supercapacitors.

The cyclic stability of supercapacitors was investigated by continuously operating the galvanostatic charge/discharge process under the voltage of 0 to 3 V at a current density of 4 A g^−1^ (Figure S6). As described in Fig. 5a, D-CNTs displayed capacitance retention of 98.3% after 3000 cycling operation due to the excellent reversibility of EDLC. However, though occurring the redox reaction and volume change of PANI, a capacitance retention of 90.2% for PANI/VA-CNTs was preserved after 3000 cycles testing where the good cycling stability was not only attributed to the stable vertical-aligned structure network (Figure S7) but also good interaction between PANI and CNTs (see the inset in Fig. 2d). The energy density was also evaluated for supercapacitors. As showed in Fig. 5b, the PANI/VA-CNTs performed a high energy density of 98.1 Wh kg^−1^, which was more than 4 times larger than D-CNTs of 22.1 Wh kg^−1^. To our knowledge, the electrochemical performance of PANI/VA-CNTs based supercapacitor could be comparable to those similar materials based supercapacitors (Table S2). It was because of the combination between CNTs and PANI, supercapacitor displayed higher electrochemical performance.

## Discussion

In this study, through taking comprehensive analysis of the results as well as the structure and electrochemical activity of electrode materials, we speculated the mechanism of the high performance supercapacitor. For the D-CNTs supercapacitor, carbon nanotubes were random bundled in the electrode, which resulted in long and tortuous pathways for charge disordered transmission and less accommodation so that the supercapacitor performed poor capacitance. However, after the disordered structure becoming vertical arrangement by electric field induction, the continuously conductive pathway could allow charges directly rapid transmission. As a result, more charges accumulating into electrode, the performance of supercapacitor was improved. Furthermore, when the high electrochemical active PANI material was electropolymerized into the carbon backbone, electrochemical performance of supercapacitor would be further enhanced due to the redox reaction bringing more charges accumulated into the electrode. Therefore, the higher electrochemical performance based on PANI/VA-CNTs nanocomposite electrode was derived from the full combination of the vertical-aligned structure and electrochemical activity.

In summary, a supercapacitor based on PANI/VA-CNTs nanocomposite electrodes has been developed through combining the structure and electrochemical activity design. The supercapacitor displays high electrochemical performance such as large specific capacitance (403.3 F g^−1^) in HClO_4_ electrolyte and good cycling stability (90.2%) and high energy density (98.1 Wh kg^−1^) in EMIBF_4_ organic electrolyte. Considering of these remarkable achievement, we believe the PANI/VA-CNTs supercapacitor will have great potential for practically portable and wearable electronic applications.

## Methods

### Preparation of CNTs film

75 mg CNTs powder was dispersed in 30 ml N, N-Dimethylacetamide solution for 30 min under 200 W horn sonication treatment (2 s on and 6 s off in an ice water bath), forming gel-like dispersion of 2.5 mg ml^−1^. The as prepared 3 ml suspension was casted on glass substrate (2.5 × 7.5 cm^2^ size) at room temperature for 5 days, obtaining loose CNTs film.

### Fabrication of PANI/VA-CNTs electrode film

The electrochemical polymerization process was carried out to fabricate PANI/VA-CNTs electrode using CHI760E Workstation with chronopotentiometry model. The CNTs film was used as working electrode, platinum plate was used as the counter electrode (20 mm × 30 mm) and Ag/AgCl was employed as the reference electrode. The electrolyte was 1 M HClO_4_ solution, containing 0.1 M aniline. The PANI/VA-CNTs was prepared at constant current of 0.5 mA cm^−2^ and the voltage was maintained at about 0.75 V for 20 min, 40 min, 60 min and 80 min. After the polymerization, the working electrode was removed from the electrolyte and rinsed using deionized water and ethanol for three times, and then dried at 80 °C for 1 day, obtaining PANI/VA-CNTs film.

### Construction of PANI/VA-CNTs supercapacitor and the characterization

The aqueous electrolyte based supercapacitor: three-electrode system was carried out where PANI/CNTs film was used as working electrode, platinum plate was used as the counter electrode (20 mm × 30 mm), Ag/AgCl was employed as the reference electrode, and electrolyte was 1 M HClO_4_ solution. The organic electrolyte based supercapacitor: two-electrode system, in which the supercapacitor was constructed by two as-prepared PANI/VA-CNTs nanocomposite electrodes separated by a porous paper of 50 μm. 1 M EMIBF_4_/PC organic electrolyte was used for supercapacitor (EMIBF_4_ was 1-eutyl-3-methylimidazolium tetrafluoroborate and PC was propylene carbonate). The D-CNTs based supercapacitor was fabricated by the same process. For supercapacitor, the CV, galvanostatic cycling and EIS were measured using CHI760E electrochemical work station. The specific capacitance of supercapacitors based on CV test was calculated using formula: C(F g^−1^) = 
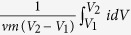
, where i (A), m (g), v (V s^−1^), V_1_ and V_2_ were the instant current, the mass of electrode, the potential scan rate and high and low potential of the CV test, respectively. The specific capacitance of supercapacitors based on galvanostatic cycle test in HClO_4_ was calculated by C(F g^−1^) = 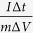, where I (A), ∆*t* (s), ∆*V* (V), and m (g) were the discharge current, the discharge current, the voltage range along discharge during the discharge process, and the mass of electrode, respectively. The specific capacitance of supercapacitors based on galvanostatic cycle test in EMIMBF_4_/PC was calculated by C(F g^−1^)=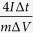, where I (A), ∆*t* (s), ∆*V* (V), and m (g) were the discharge current, the discharge current, the voltage range along discharge during the discharge process, and the total mass of the two electrode, respectively. The energy density and average power energy was calculated by E = CV^2^/8 and P = E/∆*t*, where the C (F g^−1^), V (V), and ∆*t* (s) were the cell capacitance, operated voltage and discharge time.

## Additional Information

**How to cite this article**: Wu, G. *et al*. High-performance Supercapacitors Based on Electrochemical-induced Vertical-aligned Carbon Nanotubes and Polyaniline Nanocomposite Electrodes. *Sci. Rep.* **7**, 43676; doi: 10.1038/srep43676 (2017).

**Publisher's note:** Springer Nature remains neutral with regard to jurisdictional claims in published maps and institutional affiliations.

## Supplementary Material

Supplementary Information

## References

[b1] Lu X. H., Yu M. H., Wang G. M., Tong Y. X. & Li Y. Flexible solid-state supercapacitors: design, fabrication and applications. Energ Environ Sci 7, 2160–2181 (2014).

[b2] Gao Z., Yang W. L., Wang J., Song N. N. & Li X. D. Flexible all-solid-state hierarchical NiCo2O4/porous graphene paper asymmetric supercapacitors with an exceptional combination of electrochemical properties. Nano Energy 13, 306–317 (2015).

[b3] Cong H. P., Chen J. F. & Yu S. H. Graphene-based macroscopic assemblies and architectures: an emerging material system. Chem Soc Rev 43, 7295–7325 (2014). 25065466 10.1039/c4cs00181h

[b4] Zhang Y. . Progress of electrochemical capacitor electrode materials: A review. Int J Hydrogen Energ 34, 4889–4899 (2009).

[b5] Zhai Y. P. . Carbon Materials for Chemical Capacitive Energy Storage. Adv Mater 23, 4828–4850 (2011). 21953940 10.1002/adma.201100984

[b6] Lee J. A. . Ultrafast charge and discharge biscrolled yarn supercapacitors for textiles and microdevices. Nat Commun 4, 1970 (2013). 23733169 10.1038/ncomms2970

[b7] Yang X. W., Cheng C., Wang Y. F., Qiu L. & Li D. Liquid-Mediated Dense Integration of Graphene Materials for Compact Capacitive Energy Storage. Science 341, 534–537 (2013). 23908233 10.1126/science.1239089

[b8] Ghaffari M. . High-Volumetric Performance Aligned Nano-Porous Microwave Exfoliated Graphite Oxide-based Electrochemical Capacitors. Adv Mater 25, 4879–4885 (2013). 23946189 10.1002/adma.201301243

[b9] Xu Y. X. . Holey graphene frameworks for highly efficient capacitive energy storage. Nat Commun 5, 4554 (2014). 25105994 10.1038/ncomms5554

[b10] Simon P. & Gogotsi Y. Materials for electrochemical capacitors. Nat Mater 7, 845–854 (2008). 18956000 10.1038/nmat2297

[b11] Cong H. P., Ren X. C., Wang P. & Yu S. H. Flexible graphene-polyaniline composite paper for high-performance supercapacitor. Energ Environ Sci 6, 1185–1191 (2013).

[b12] Yun J., Kim D., Lee G. & Ha J. S. All-solid-state flexible micro-supercapacitor arrays with patterned graphene/MWNT electrodes. Carbon 79, 156–164 (2014).

[b13] Xu J., Wang K., Zu S.-Z., Han B.-H. & Wei Z. Hierarchical nanocomposites of polyaniline nanowire arrays on graphene oxide sheets with synergistic effect for energy storage. Acs Nano 4, 5019–5026 (2010). 20795728 10.1021/nn1006539

[b14] Kaempgen M., Chan C. K., Ma J., Cui Y. & Gruner G. Printable Thin Film Supercapacitors Using Single-Walled Carbon Nanotubes. Nano Lett 9, 1872–1876 (2009). 19348455 10.1021/nl8038579

[b15] Nardecchia S., Carriazo D., Ferrer M. L., Gutierrez M. C. & del Monte F. Three dimensional macroporous architectures and aerogels built of carbon nanotubes and/or graphene: synthesis and applications. Chem Soc Rev 42, 794–830 (2013). 23160635 10.1039/c2cs35353a

[b16] Pan S. W., Ren J., Fang X. & Peng H. S. Integration: An Effective Strategy to Develop Multifunctional Energy Storage Devices. Adv Energy Mater 6, 1501867 (2016).

[b17] Wang B. J. . Fabricating Continuous Supercapacitor Fibers with High Performances by Integrating All Building Materials and Steps into One Process. Adv Mater 27, 7854–7860 (2015). 26488334 10.1002/adma.201503441

[b18] Wang Y. . High-Performance Flexible Solid-State Carbon Cloth Supercapacitors Based on Highly Processible N-Graphene Doped Polyacrylic Acid/Polyaniline Composites. Sci Rep-Uk 6, 12883 (2016). 10.1038/srep12883 PMC4756666 26883179

[b19] Hu N. . Three-dimensional skeleton networks of graphene wrapped polyaniline nanofibers: an excellent structure for high-performance flexible solid-state supercapacitors. Sci Rep-Uk 6, 19777 (2016). 10.1038/srep19777 PMC4726342 26795067

[b20] Niu Z. Q. . Compact-designed supercapacitors using free-standing single-walled carbon nanotube films. Energ Environ Sci 4, 1440–1446 (2011).

[b21] Pham D. T. . Carbon Nanotube-Bridged Graphene 3D Building Blocks for Ultrafast Compact Supercapacitors. Acs Nano 9, 2018–2027 (2015). 25643138 10.1021/nn507079x

[b22] Zhang C. G. . Splitting of a Vertical Multiwalled Carbon Nanotube Carpet to a Graphene Nanoribbon Carpet and Its Use in Supercapacitors. Acs Nano 7, 5151–5159 (2013). 23672653 10.1021/nn400750n

[b23] Zhou Y. . Advanced asymmetric supercapacitor based on conducting polymer and aligned carbon nanotubes with controlled nanomorphology. Nano Energy 9, 176–185 (2014).

[b24] Meng C. Z., Liu C. H., Chen L. Z., Hu C. H. & Fan S. S. Highly Flexible and All-Solid-State Paper like Polymer Supercapacitors. Nano Lett 10, 4025–4031 (2010). 20831255 10.1021/nl1019672

[b25] Hyder M. N. . Layer-by-Layer Assembled Polyaniline Nanofiber/Multiwall Carbon Nanotube Thin Film Electrodes for High-Power and High-Energy Storage Applications. Acs Nano 5, 8552–8561 (2011). 21981582 10.1021/nn2029617

[b26] Liao Y. Z., Li X. G., Hoek E. M. V. & Kaner R. B. Carbon nanotube/polyaniline nanofiber ultrafiltration membranes. J Mater Chem A 1, 15390–15396 (2013). 10.1039/c3nr00441d 23525119

[b27] Wu Q., Xu Y. X., Yao Z. Y., Liu A. R. & Shi G. Q. Supercapacitors Based on Flexible Graphene/Polyaniline Nanofiber Composite Films. Acs Nano 4, 1963–1970 (2010). 20355733 10.1021/nn1000035

[b28] Zhang Z. T. . Superelastic Supercapacitors with High Performances during Stretching. Adv Mater 27, 356–362 (2015). 25424189 10.1002/adma.201404573

[b29] Lin H. J. . Conducting polymer composite film incorporated with aligned carbon nanotubes for transparent, flexible and efficient supercapacitor. Sci Rep-Uk 3, 1353 (2013). 10.1038/srep01353 PMC3582998 23443325

[b30] Niu Z. Q. . A “skeleton/skin” strategy for preparing ultrathin free-standing single-walled carbon nanotube/polyaniline films for high performance supercapacitor electrodes. Energ Environ Sci 5, 8726–8733 (2012).

[b31] Ge D. T. . Foldable supercapacitors from triple networks of macroporous cellulose fibers, single-walled carbon nanotubes and polyaniline nanoribbons. Nano Energy 11, 568–578, (2015).

[b32] Castellano R. J. . Electrokinetics of scalable, electric-field-assisted fabrication of vertically aligned carbon-nanotube/polymer composites. J Appl Phys 117, 214306 (2015).

[b33] Wang H., Zhang H., Zhao W., Zhang W. & Chen G. Preparation of polymer/oriented graphite nanosheet composite by electric field-inducement. Composites Science and Technology 68, 238–243 (2008).

[b34] Bradley J. C. . Creating electrical contacts between metal particles using directed electrochemical growth. Nature 389, 268–271 (1997).

[b35] Nguyen T. T., Grosberg A. Y. & Shklovskii B. I. Screening of a charged particle by multivalent counterions in salty water: Strong charge inversion. Journal of Chemical Physics 113, 1110–1125 (2000).

[b36] Wu G. . Ordered and Active Nanochannel Electrode Design for High-Performance Electrochemical Actuator. Small 12, 4986–4992 (2016). 27119424 10.1002/smll.201600973

[b37] Yao Q., Wang Q., Wang L. & Chen L. Abnormally enhanced thermoelectric transport properties of SWNT/PANI hybrid films by the strengthened PANI molecular ordering. Energy Environ. Sci. 7, 3801–3807 (2014).

[b38] Liu Q. . Synergistic effect of a r-GO/PANI nanocomposite electrode based air working ionic actuator with a large actuation stroke and long-term durability. J. Mater. Chem. A 3, 8380–8388 (2015).

[b39] Zhang J. T., Zhao Z. H., Xia Z. H. & Dai L. M. A metal-free bifunctional electrocatalyst for oxygen reduction and oxygen evolution reactions. Nat Nanotechnol 10, 444–452 (2015). 25849787 10.1038/nnano.2015.48

[b40] Wu G. . An interface nanostructured array guided high performance electrochemical actuator. J. Mater. Chem. A, 2, 16836–16841 (2014).

[b41] Bisquert J. Analysis of the kinetics of ion intercalation - Ion trapping approach to solid-state relaxation processes. Electrochim Acta 47, 2435–2449 (2002).

