# Supplementary material for: High-performance Supercapacitors Based on Electrochemical-induced Vertical-aligned Carbon Nanotubes and Polyaniline Nanocomposite Electrodes
Source: Sci Rep. 2017 Mar 8;7:43676. doi: 10.1038/srep43676 (PMC5341108; doi:10.1038/srep43676)
Supplement: Supplementary Information [file srep43676-s1.pdf]

High-performance Supercapacitors Based on Electrochemical-induced Vertical-aligned Carbon Nanotubes and Polyaniline Nanocomposite Electrodes

Guan Wu[1], Pengfeng Tan[1], Dongxing Wang[2], Zhe Li[2], Lu Peng[1], Ying Hu[3], Caifeng Wang[2], Wei Zhu[2], Su Chen[1]* and Wei Chen[2]*

[1]The State Key Laboratory of Materials-Oriented Chemical Engineering and College of Chemistry and Chemical Engineering, Nanjing Tech University, Nanjing 210009, P. R. China.
[2]i-Lab, Suzhou Institute of Nano-tech and Nano-bionics, Chinese Academy of Sciences, Suzhou 215123, P. R. China.
[3]Institute of Industry and Equipment Technology, Hefei University of Technology, Hefei, Anhui 230009, P. R. China.

*Corresponding author: chensu@njtech.edu.cn and wchen2006@sinano.ac.cn

Supplementary Table 1| EIS molding data. Parameter values from curve-fitting of the impedance results shown in Fig. 4d by using the equivalent circuit described in inset of Fig. 4d.

	R_0/Ω	C_1/mF s^{n_1-1}	n_1	R_1/Ω	Z_w/Ω	C_2/mF	n_2
D-CNTs	15.3	0.14	0.82	2.32	2.15	2.74	0.84
PANI/VA-CNTs	9.6	0.94	0.87	0.75	0.43	18.3	0.90

Supplementary Table 2| Specific capacitance and energy density values of different carbon-based materials for supercapacitors.

Materials	Capacitance	Energy density	References
PPY/SWCNTs	200 F g^{-1} in KCl solution		1
PEDOT/functionalized SWCNTs	210 F g^{-1} in KCl solution		2
PANI/CNTs	350 F g^{-1} in H_2SO_4/PVA	7.1 Wh kg^{-1}	3
SWCNT/PANI array	410 F g^{-1} in H_2SO_4 solution	26.6 Wh kg^{-1}	4
Layer-by-Layer Assembled PANI/MWCNTs	238 F cm^{-3} in $LiPF_6$	220 Wh L^{-1}	5
PANI/SWCNTs	236 F g^{-1} in LiClO4	131 Wh kg^{-1}	6
Aligned-MWCNTs/PANI	223 F g^{-1} in H_3PO_4/PVA		7
PEDOT/MWCNTs yarns	179 F cm^{-3} in H_3PO_4/PVA	1.4 mWh cm^{-1}	8
CNTs/PPy	184 F g^{-1} in KCl solution		9
Aligned-CNTs/PEDOT	205 F g^{-1} in $BIMBF_4$	82.8 Wh L^{-1}	10
CNTs/PANI hydrogel	315 F g^{-1} in H_3PO_4/PVA		11
PANI/CNTs nanofibers	385 in acid solution		12
PANI/rGO	211 F g^{-1} in H_2SO_4/PVA	29.3 Wh kg^{-1}	13
Nitrogen-doped carbon/PANI	134 F g^{-1} in Na_2SO_4	60.3 Wh kg^{-1}	14
Our work	403.3 F/g in $HClO_4$<br>314.6 F/g in $EIMBF_4$	98.1 Wh kg^{-1}	

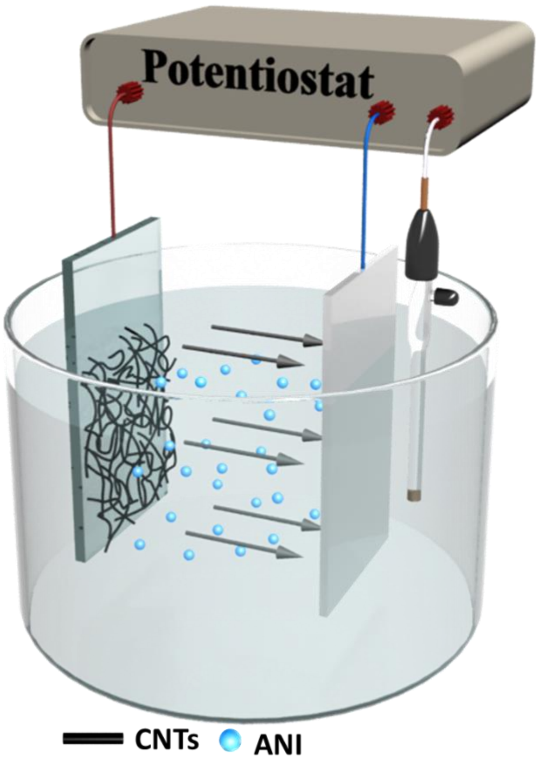

Supplementary Fig. 1. Schematic illustration of the fabrication of PANI/VA-CNTs in the three-electrode system of electrochemical polymerization.

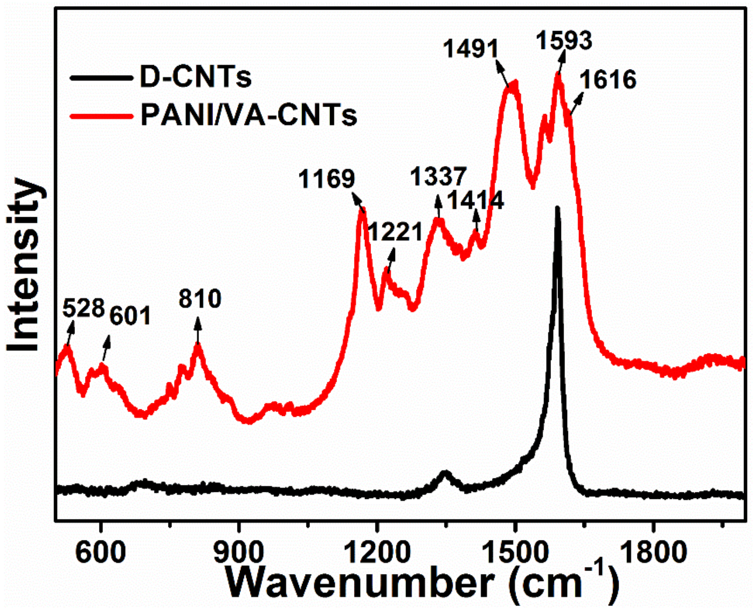

Supplementary Fig. 2. Raman spectrum of D-CNTs and PANI/VA-CNTs films.

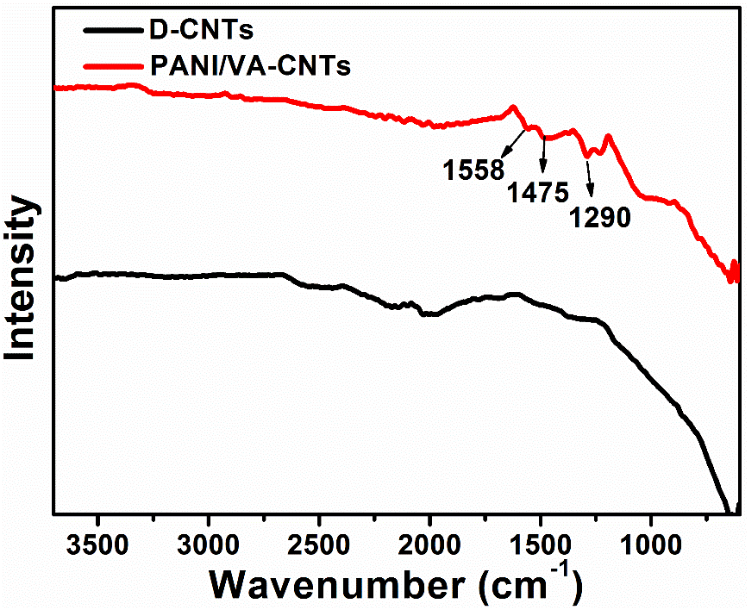

Supplementary Fig. 3. FTIR spectrum of D-CNTs and PANI/VA-CNTs films.

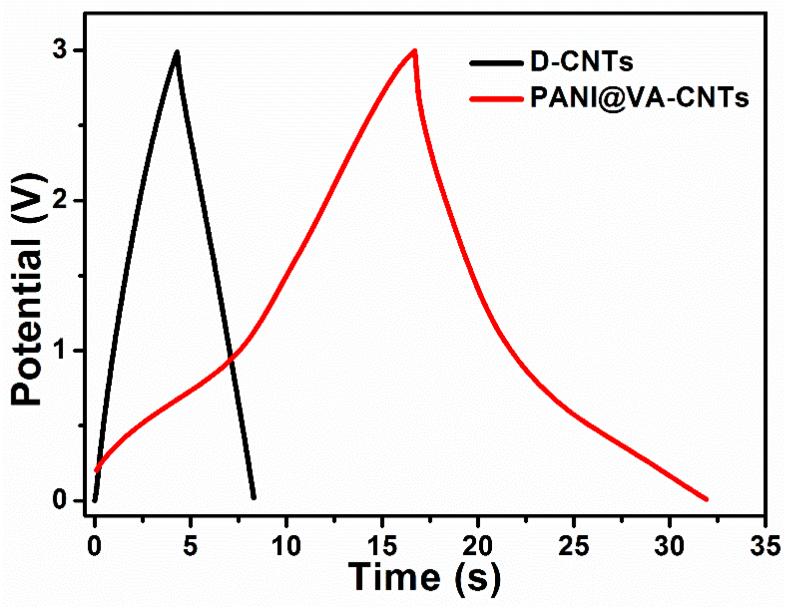

Supplementary Fig. 4. Galvanostatic charge/discharge curves at a current density of 10 A g^{-1}.

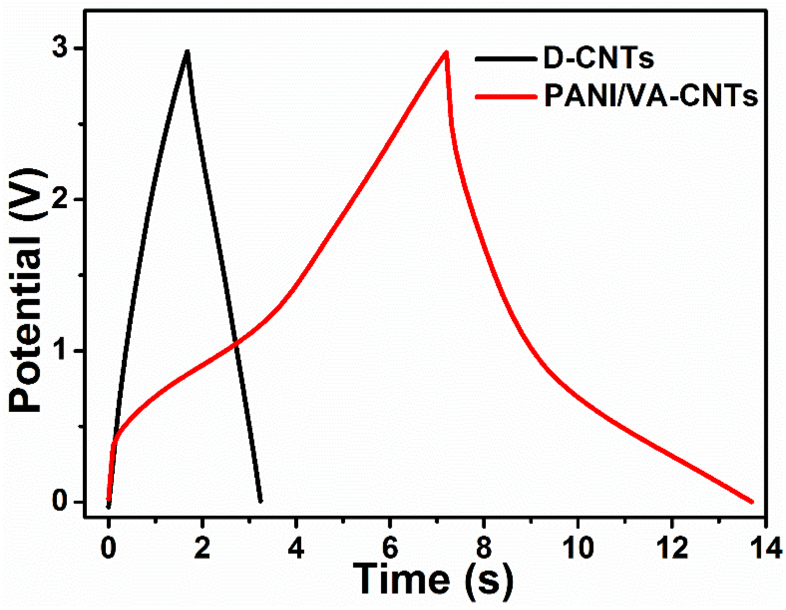

Supplementary Fig. 5. Galvanostatic charge/discharge curves at the current density of 20 A g^{-1}.

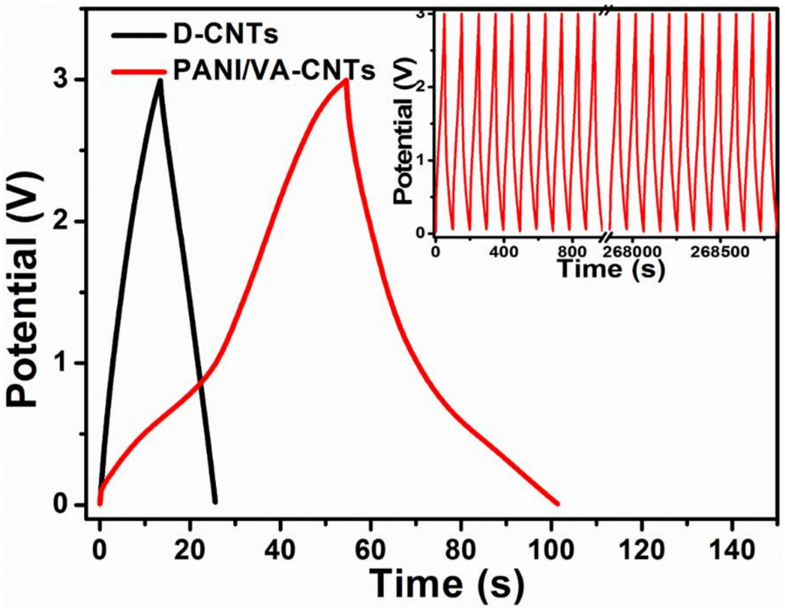

Supplementary Fig. 6. The continuous stability test of PANI/VA-CNTs at the current density of 4 A g^{-1}.

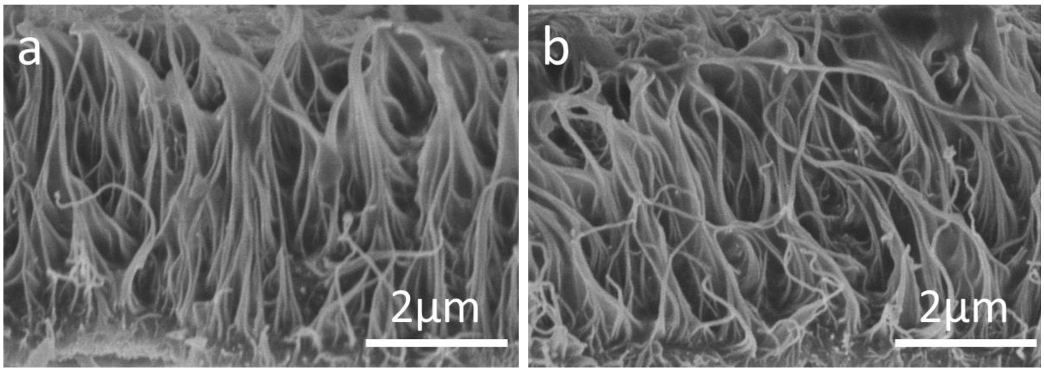

Supplementary Fig. 7. The SEM image of PANI/VA-CNTs before (**a**) and after (**b**) 3000 cycling tests.

References

1 Wang, J., Xu, Y., Chen, X. & Sun, X. Capacitance properties of single wall carbon nanotube/polypyrrole composite films. *Composites Science and Technology* **67**, 2981-2985 (2007).

2 Wang, J., Xu, Y., Sun, X., Li, X. & Du, X. Electrochemical capacitance of the composite of poly (3,4-ethylenedioxythiophene) and functionalized single-walled carbon nanotubes. *Journal of Solid State Electrochemistry* **12**, 947-952 (2008).

3 Meng, C., Liu, C., Chen, L., Hu, C. & Fan, S. Highly Flexible and All-Solid-State Paper like Polymer Supercapacitors. *Nano Lett* **10**, 4025-4031 (2010).

4 Wang, K., Zhao, P., Zhou, X., Wu, H. & Wei, Z. Flexible supercapacitors based on cloth-supported electrodes of conducting polymer nanowire array/SWCNT composites. *J Mater Chem* **21**, 16373-16378 (2011).

5 Hyder, M. N. *et al.* Layer-by-Layer Assembled Polyaniline Nanofiber/Multiwall Carbon Nanotube Thin Film Electrodes for High-Power and High-Energy Storage Applications. *Acs Nano* **5**, 8552-8561 (2011).

6 Niu, Z. *et al.* A "skeleton/skin" strategy for preparing ultrathin free-standing single-walled carbon nanotube/polyaniline films for high performance supercapacitor electrodes. *Energ Environ Sci* **5**, 8726-8733 (2012).

7 Lin, H. *et al.* Conducting polymer composite film incorporated with aligned carbon nanotubes for transparent, flexible and efficient supercapacitor. *Sci Rep-Uk* **3**, 1353 (2013).

8 Lee, J. A. *et al.* Ultrafast charge and discharge biscrolled yarn supercapacitors for textiles and microdevices. *Nat Commun* **4**, 2970 (2013).

9 Liu, F. *et al.* Fabrication of Carbon Nanotubes/Polypyrrole/Carbon Nanotubes/Melamine Foam for Supercapacitor. *Journal of Applied Polymer Science* **131**, 39779 (2014).

10 Zhou, Y. *et al.* Advanced asymmetric supercapacitor based on conducting polymer and aligned carbon nanotubes with controlled nanomorphology. *Nano Energy* **9**, 176-185 (2014).

11 Xiang, X. *et al.* Smart and flexible supercapacitor based on a porous carbon nanotube film and polyaniline hydrogel. *Rsc Advances* **6**, 24946-24951 (2016).

12 Simotwo, S. K., DelRe, C. & Kalra, V. Supercapacitor Electrodes Based on High-Purity Electrospun Polyaniline and Polyaniline-Carbon Nanotube Nanofibers. *Acs Applied Materials & Interfaces* **8**, 21261-21269 (2016).

13 Hu, N. *et al.* Three-dimensional skeleton networks of graphene wrapped polyaniline nanofibers: an excellent structure for high-performance flexible solid-state supercapacitors. *Sci Rep-Uk* **6**,

19777 (2016).

14 Yu, P. *et al.* A Novel Sustainable Flour Derived Hierarchical Nitrogen-Doped Porous Carbon/Polyaniline Electrode for Advanced Asymmetric Supercapacitors. *Adv Energy Mater* **6**, 1601111 (2016).
